# Editorial: EBV-Associated Carcinomas: Presence, Role, and Prevention Strategies

**DOI:** 10.3389/fonc.2018.00528

**Published:** 2018-11-16

**Authors:** Ala-Eddin Al Moustafa, Hussain G. Ahmed, Gerburg Wulf, Ali A. Sultan

**Affiliations:** ^1^College of Medicine, Qatar University, Doha, Qatar; ^2^Biomedical Research Centre, Qatar University, Doha, Qatar; ^3^Oncology Department, McGill University, Montreal, QC, Canada; ^4^College of Medicine, University of Hail, Ha'il, Saudi Arabia; ^5^Beth Israel Deaconess Medical Center, Harvard Medical School, Boston, MA, United States; ^6^Weill Cornell Medicine-Qatar, Ar-Rayyan, Qatar

**Keywords:** Epstein-Barr virus, cervical cancer, breast cancer, epithelial to mesenchymal transition, EBV vaccine

This special issue addresses an important topic related to the role of Epstein-Barr virus (EBV) in human carcinomas initiation and progression, which is one of the most common viral infections worldwide. Today, the relationship between EBV infection and several types of human lymphomas is clearly established, including Hodgkin and Burkitt's lymphoma; meanwhile, it was recently pointed out that EBV is present in nasopharyngeal carcinomas as well as other epithelial cancers (1). EBV is ubiquitous human herpesvirus 4, its genome codes more than 85 proteins of which only few are well-understood; More specifically, six nuclear antigens (EBNA: 1, 2, 3A, 3B, 3C, and LP); three latent membrane proteins/genes (LMP: 1, 2A, 2B) as well as small non-polyadenylated RNAs, EBERs 1 and 2 in addition to few microRNAs have been identified so far, as key regulators, of the oncogenic activity of this virus (2, 3). Present estimates indicate that EBV causes 200,000 new cancer cases annually, accounting for ~2% of cancers worldwide (Cancer Research UK). On the other hand, it is important to emphasize that recent investigations have revealed the possible involvement of EBV in other cancers such as cervical, gliomas, and breast, which are highlighted in this issue.

This topic comprises nine manuscripts that cover the involvement of EBV in human carcinomas. Within this special issue the reader will become familiar with the most studied EBV oncoproteins and their role in carcinogenesis. More specifically, El-Sharkawy et al. discuss the role of LMP1 and LMP2A as well as EBV nuclear antigens (EBNAs) in EBV persistence and latency infection. Moreover, the authors highlight the roles of these oncoproteins in activating different signal transduction pathways which are critical for cell growth and survival and can present a potential therapeutic target for EBV-associated cancers. While, Smatti et al. provide a review of EBV epidemiology and genetic variability of the LMP1 oncoprotein. They also detail the most recent findings of EBV seroprevalence and viremia studies specially in healthy blood donors as a highly prevalent way of transmitting oncoviruses including EBV.

An up-to-date account of the role of EBV in several known carcinomas are also covered, starting with the presence of EBV in cervical cancer in a review paper by Vranic et al. then, in gliomas by Akhtar et al. breast cancer by Abdallah et al. as well as head and neck (HN) cancer by Fernandes et al. More specifically, the reader will perceive the controversy of this virus being associative, causative, or an experimental artifact in clinical literature, and the conflicting data results on the presence of EBV in gliomas (Akhtar et al.). In addition, the important issue of immunological aspects underlying the infection by this oncovirus and the use of immunotherapeutic interventions as a potential modality for targeting EBV-associated HN cancers (Fernandes et al.). Moreover, according to Abdallah et al. there is no doubt in the pathogenic role of EBV in breast cancer, as their pilot genome-wide methylome study in breast cancer samples from Sudan identified clear genetic differences between primary tumor samples and adjacent normal tissues from the same patients. Differential methylation analysis revealed developmental and viral pathways dysregulations, including EBV infection, that can be used for targeted therapies of breast cancer. On the other hand, possible interactions between EBV and other oncoviruses, such as HPVs are touched upon by Vranic et al. implicating the role of co-infection in the full development of cervical cancer pathogenesis. EBV and HPVs co-presence in cervical cancer was confirmed in the original research paper presented by Al-Thawadi et al. that investigated this aspect in cervical cancer samples from Syrian women. Based on their study, EBV and high-risk HPVs are associated with highly aggressive cancer phenotype in human cervical cancer, which begs for extensive research into the cooperation between these two common oncoviruses.

The cooperative role of EBV and high-risk HPVs is fully addressed by Cyprian et al. detailing their possible role in the initiation and/or amplification of epithelial to mesenchymal transition (EMT), which is the hallmark of cancer progression. The authors propose that this cooperation can occur via β-catenin, JAK/STAT/SRC, PI3k/Akt/mTOR, and/or RAS/MEK/ERK signaling pathways as both EBV and HPVs share these paths.

Finally, Khan et al. present an original research paper on the role of Skp2 and its ubiquitin-proteasome pathway in HN carcinomas. They found that treatment of HN cancer cells with curcumin or transfection of small interfering RNA of Skp2, causes down-regulation of Skp2 in HPV+, HPV– cells. Additionally, treatment with curcumin induced apoptosis via mitochondrial pathway and activation of caspases. While, co-treatment of HN cancer cells with curcumin and cisplatin also inhibited cell viability and apoptotic effects. This is an interesting finding since an important part of HN cancers, the majority of oral cancers, are positive for EBV; thus, we believe that this kind of study can be extended to EBV or EBV/HPV positive human carcinomas.

This collection of manuscripts addresses important health gaps with regards to the role of EBV infection in human carcinomas which are of global interest, as increasing number of malignancies are linked to EBV infection, and the majority of these cases occur in developing countries. Therefore, studies combined with awareness campaigns that emphasize the role of simple hygienic measures as a cancer prevention strategy; in addition to understanding the importance of the upcoming EBV vaccine may play a crucial role in the prevention and reduction of EBV related cancers (4). On the other hand, more investigations, on cellular and molecular level, are necessary to elucidate the oncogenic role of EBV in human carcinomas. More specifically, crosstalk between EBV and other oncoviruses such as high-risk HPVs is an important topic that should be taken into consideration since it is well-know that oncogenesis is a complex process involving several oncogenes (c-onc and v-onc) as well as other factors.

Finally, we are thankful to the authors of these manuscripts who have responded and enriched the topic with their valuable contributions. The findings of these manuscripts are interesting and contribute to our understanding of the complex role of EBV in human carcinomas.

## Author contributions

A-EA, HA, GW, and AS edited the paper. A-EA wrote the paper from conception to its finalized form.

### Conflict of interest statement

The authors declare that the research was conducted in the absence of any commercial or financial relationships that could be construed as a potential conflict of interest.
